# Culture Conditions Affect Cardiac Differentiation Potential of Human Pluripotent Stem Cells

**DOI:** 10.1371/journal.pone.0048659

**Published:** 2012-10-31

**Authors:** Marisa Ojala, Kristiina Rajala, Mari Pekkanen-Mattila, Marinka Miettinen, Heini Huhtala, Katriina Aalto-Setälä

**Affiliations:** 1 Institute of Biomedical Technology, University of Tampere, Tampere, Finland; 2 BioMediTech, University of Tampere, Tampere, Finland; 3 School of Health Sciences, University of Tampere, Tampere, Finland; 4 Heart Center, Tampere University Hospital, Tampere, Finland; University of Melbourne, Australia

## Abstract

Human pluripotent stem cells (hPSCs), including human embryonic stem cells (hESCs) and human induced pluripotent stem cells (hiPSCs), are capable of differentiating into any cell type in the human body and thus can be used in studies of early human development, as cell models for different diseases and eventually also in regenerative medicine applications. Since the first derivation of hESCs in 1998, a variety of culture conditions have been described for the undifferentiated growth of hPSCs. In this study, we cultured both hESCs and hiPSCs in three different culture conditions: on mouse embryonic fibroblast (MEF) and SNL feeder cell layers together with conventional stem cell culture medium containing knockout serum replacement and basic fibroblast growth factor (bFGF), as well as on a Matrigel matrix in mTeSR1 medium. hPSC lines were subjected to cardiac differentiation in mouse visceral endodermal-like (END-2) co-cultures and the cardiac differentiation efficiency was determined by counting both the beating areas and Troponin T positive cells, as well as studying the expression of *OCT-3/4*, mesodermal *Brachyury T* and *NKX2.5* and endodermal *SOX-17* at various time points during END-2 differentiation by q-RT-PCR analysis. The most efficient cardiac differentiation was observed with hPSCs cultured on MEF or SNL feeder cell layers in stem cell culture medium and the least efficient cardiac differentiation was observed on a Matrigel matrix in mTeSR1 medium. Further, hPSCs cultured on a Matrigel matrix in mTeSR1 medium were found to be more committed to neural lineage than hPSCs cultured on MEF or SNL feeder cell layers. In conclusion, culture conditions have a major impact on the propensity of the hPSCs to differentiate into a cardiac lineage.

## Introduction

Human pluripotent stem cells (hPSCs) include human embryonic stem cells (hESCs) and human induced pluripotent stem cells (hiPSCs). hPSCs are able to self-renew and to differentiate into any human cell type; therefore, they can be used as a cell model to study embryology and disease pathophysiology. hPSCs have additional utility in drug screening applications and as a cell source for regenerative medicine in the future. Since the first derivation of a hESC line in 1998 on a mouse embryonic fibroblast (MEF) feeder cell layer [1], many hPSC culture methods based on different human feeder cell layers [2], [3], autologous feeder cells [4], [5], feeder cell-free [6]–[10] and suspension culture techniques [11] have been developed and described. Feeder cells provide appropriate cell contacts, various growth factors and extracellular matrix (ECM) proteins that are required for the undifferentiated growth of hPSCs. Animal-derived feeder cells and other animal components used in hPSC culture conditions contain animal proteins and other nonhuman molecules which could be transmitted to hPSCs during culture [12], [13]. Because the ultimate aim of hPSC research is to use the cells in regenerative medicine applications, culture conditions are being optimized in the xeno-free direction. In addition, culturing feeder cells is very laborious and time-consuming, and for the regenerative medicine applications, a large number of hPSCs are needed. Therefore the research is aiming at developing both xeno- and feeder cell-free cultures. In feeder cell-free culture methods, the feeder cells are replaced by Matrigel, which is a basement membrane extract from mouse tumor cells, or by ECM proteins such as laminin, collagen and fibronectin [6]–[10]. Despite the tremendous effort made to optimize hPSC culture conditions, a universal and reliable, xeno- and feeder-free culture method remains to be discovered.

Each individual hESC line has a unique gene expression profile [14], [15] and thus the self-renewal and differentiation capabilities vary among the different cell lines [16], [17]. According to recent reports, hiPSC lines are even more variable and more prone to genomic alterations than hESCs [18]–[21]. In addition to the differences among individual hPSC lines, differences in culture conditions also have a considerable influence on the gene expression profile and subsequent characteristics of hPSCs. For example, serum- and feeder cell-free culture conditions, as well as the processes of enzymatic passaging and culturing of hPSCs in physiological normoxia (2%), have been found to alter the gene expression profile and epigenome of hPSCs [22]–[24].

Efficient cardiac differentiation methods are needed to produce large numbers of human cardiomyocytes for research purposes and for future regenerative medicine applications. Due to the unique gene expression profile and variable differentiation potential of each individual hPSC line, it may be challenging to develop a universal cardiac differentiation protocol that is applicable and efficient for all or even the majority of hPSC lines. Thus, it has been proposed that individual hPSC lines may require optimization of the cardiac differentiation conditions [25]. We hypothesized that in addition to cardiac differentiation methods, the hPSC culture method, in which cell lines are cultured prior to differentiation, may also have a significant impact on the cardiac differentiation potential of individual hPSC lines. In this study, the impact of three different culture methods for cardiac differentiation of hPSCs were compared: MEF and SNL feeder cell layers combined with conventional stem cell culture medium containing knockout serum replacement (ko-SR) and basic fibroblast growth factor (bFGF), and a Matrigel matrix combined with commercial mTeSR1 medium.

## Results

### The morphology of the pluripotent stem cell colonies varies with the culture conditions

**Figure 1:**
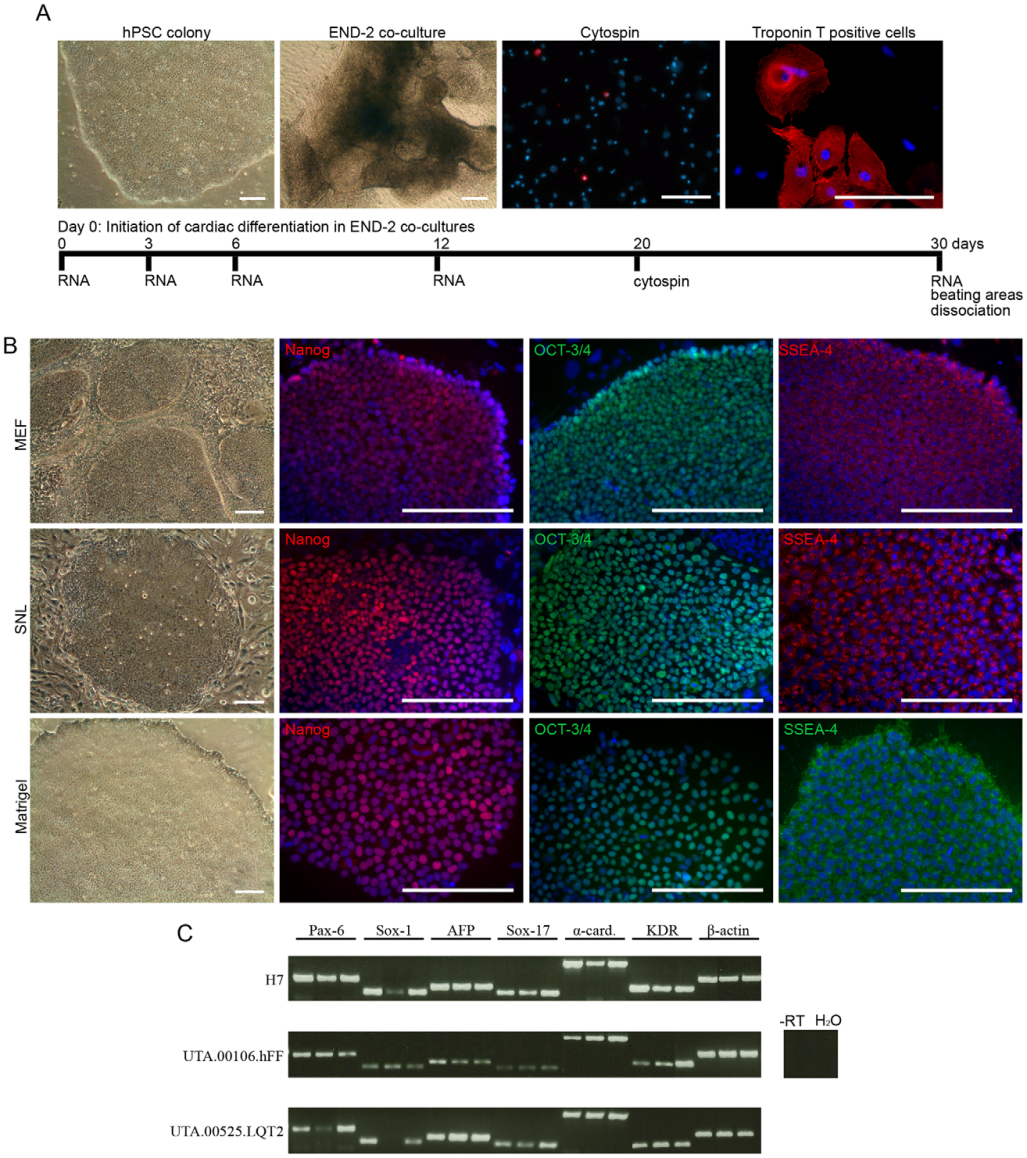
hPSCs were cultured with three different culture methods. A) Schematic presentation of cardiac differentiation in END-2 co-culture and the experimental design. hPSC  =  human pluripotent stem cell, END-2 =  mouse visceral endodermal-like cells. Scale bars, 200 μm. B) All five hPSC lines cultured on MEF and SNL feeder cell layers in conventional stem cell culture medium and on Matrigel in mTeSR1 medium at least for 14 passages formed undifferentiated colonies, which expressed pluripotency markers Nanog, OCT-3/4 and SSEA-4. Representative images of UTA.00112.hFF (phase contrast microscope images) and H7 (immunofluorescence images) cell lines are presented. Scale bars, 200 μm. C) H7, UTA.00106.hFF and UTA.00525.LQT2 cell lines cultured in all three culture conditions (in figure: first band MEF, second band SNL, third band Matrigel) formed embryoid bodies (EBs) expressing markers from all germ layers: ectoderm (*PAX-6* and *SOX-1*), endoderm (*AFP* and *SOX-17*) and mesoderm (*α-cardiac actin* and *KDR*).

In this study, a single hESC line (H7) and four hiPSC lines (UTA.00112.hFF, UTA.04602.WT, UTA.00525.LQT2 and UTA.00106.hFF) were cultured with three different culture methods: MEF and SNL feeder cell layers combined with conventional stem cell culture medium and Matrigel matrix combined with mTeSR1 medium, and subjected into cardiac differentiation in mouse visceral endodermal-like cell (END-2) co-cultures. The experimental design is presented in Figure 1A. hPSC lines cultured in different conditions were characterized by the morphology of the colonies, immunocytochemical staining and embryoid body (EB) formation (Figure 1). All cell lines attached well to both MEF and SNL feeder cell layers and to Matrigel matrix after passaging, while the morphology of the colonies varied under different culture conditions. On MEF feeder cells, the colonies were thick and small, while on SNL feeders and on Matrigel the colonies were large and thin and thus needed to be passaged more often than hPSCs cultured on MEF feeders (Figure 1B). It was difficult to adapt the H7 and UTA.00112.hFF cell lines to the Matrigel matrix in mTeSR1 medium because of the spontaneous neural differentiation. The H7 cell line was lost once and the adaptation had to be started from the beginning, and the UTA.00112.hFF cell line had to be cultured for 12 passages on Matrigel before enough cells were generated for the cardiac differentiation. However, all hPSC lines cultured at least for 14 passages in all three conditions expressed markers typical of undifferentiated hPSCs (Nanog, octamer-binding transcription factor 3/4 (OCT-3/4) and stage-specific embryonic antigen 4 (SSEA-4)), which were detected by immunocytochemical staining (Figure 1B). The pluripotency of the H7, UTA.00106.hFF and UTA.00525.LQT2 cell lines, cultured in all three conditions, was verified by EB formation and the EBs expressed at least one marker from all three germ layers (Figure 1C).

### Pluripotent stem cells from all culture conditions differentiate into cardiomyocytes

hPSC lines cultured in all three culture conditions were differentiated into cardiomyocytes at least twice in END-2 co-cultures (See Table 1 for the number of independent experiments). hPSCs from MEF feeder cell layers formed uniform structures on END-2 cells, while hPSCs from SNL feeder cells formed irregular structures consisting of both cystic structures and uniform areas. Cells, which were cultured on Matrigel in mTeSR1 medium prior the differentiation, formed large, thick and uniform structures on END-2 cells (data not shown). Interestingly, microtubule associated protein 2 (MAP-2) expressing neural-like cells and bundles of nerve fibers were occasionally observed in END-2 co-cultures of all cell lines previously cultured on Matrigel in mTeSR1 medium. These structures were not detected in hPSCs originally cultured on MEF or SNL feeder cell layers.

**Table 1:** Cell lines and their passages used in this study.

		Passage during differentiation experiments							
	Culture								
Cell line	condition	1.	2.	3.	4.	5.	6.	TNE	TNW
H7	MEF	44	51	55	47	51	57	6	105
H7	SNL	44(6)*	53(15)	–	49(6)	53(10)	63(20)	5	67
H7	Matrigel	50(6)	59(15)	–	48(5)	53(10)	60(17)	5	89
UTA.00112.hFF	MEF	–	–	–	15	18	22	3	48
UTA.00112.hFF	SNL	–	–	–	16(5)	21(10)	25(14)	3	42
UTA.00112.hFF	Matrigel	–	–	–	23(12)	26(15)	29(18)	3	54
UTA.04602.WT	MEF	–	–	–	35	40	44	3	51
UTA.04602.WT	SNL	–	–	–	37(5)	48(16)	52(20)	3	46
UTA.04602.WT	Matrigel	–	–	–	37(5)	47(15)	51(19)	3	45
UTA.00525.LQT2	MEF	45	33	37	–	–	–	3	36
UTA.00525.LQT2	SNL	44(6)	53(15)	–	–	–	–	2	18
UTA.00525.LQT2	Matrigel	44(6)	53(15)	–	–	–	–	2	30
UTA.00106.hFF	MEF	24	30	34	–	–	–	3	40
UTA.00106.hFF	SNL	23(6)	32(15)	–	–	–	–	2	25
UTA.00106.hFF	Matrigel	23(6)	32(15)	–	–	–	–	2	31

* The passages, indicating for how long hPSCs were cultured in SNL and Matrigel conditions, are given in parenthesis.

TNE  =  Total number of experiments.

TNW  =  Total number of wells from which the beating areas were counted.

**Figure 2:**
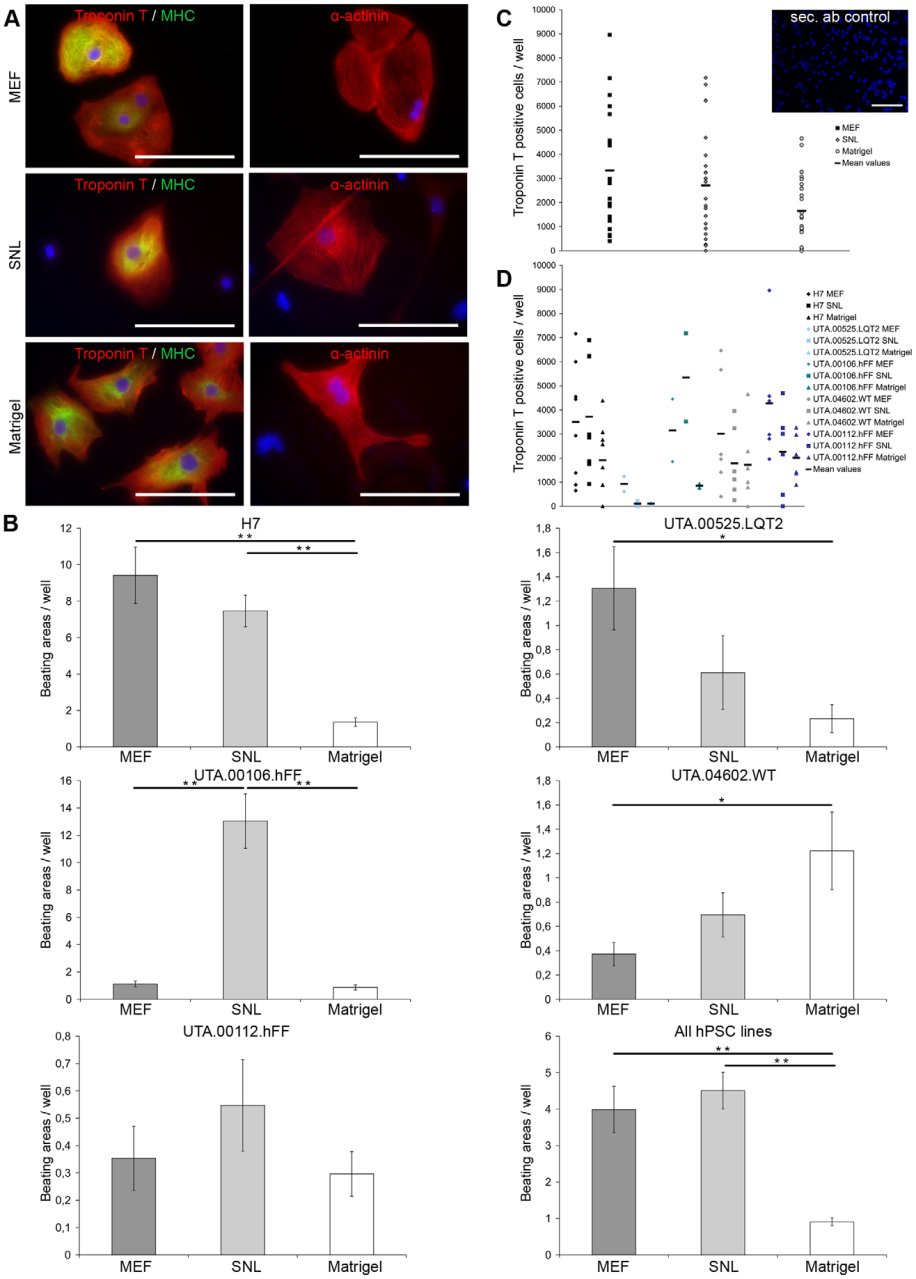
All hPSC lines cultured with different culture methods differentiated into cardiomyocytes with varying efficiencies. A) Cardiomyocytes derived from hPSCs cultured in all three culture conditions expressed Troponin T, myosin ventricular heavy chain α/β (MHC) and α-actinin. Representative images from H7 and UTA.00112.hFF cell lines. Scale bars, 200 μm. B) The columns show the average amount of beating areas in one well with different hPSC lines cultured in all three culture conditions. The number of wells from which beating areas were counted in different conditions for each cell line can be found in Table 1. In figure where all hPSC lines are collected into one histogram, the columns show the average amount of beating areas in one well of all cell lines (MEF n = 280, SNL n = 198, Matrigel n = 249). Error bars show the standard error of the mean (SEM). ** p<0.01, * p<0.05. Representative image of secondary antibody control is from H7 cell line cultured on Matrigel prior differentiation. Scale bar, 200 μm. C) Scatter plot show the amount of Troponin T positive cells in one well of all hPSC lines collected together in different conditions. The amount of Troponin T positive cells was significantly higher on MEF feeder cell layers than on Matrigel (p = 0.012). D) The scatter plot show the amount of Troponin T positive cells in one well separately for each cell line cultured in all three culture conditions.

hPSC lines cultured in all three culture conditions differentiated into cardiomyocytes and expressed Troponin T, myosin ventricular heavy chain α/β (MHC) and α-actinin (Figure 2A). Differentiation efficiency was evaluated by determining the amount of Troponin T positive cells in cytospin experiments on day 20 and by determining the number of beating areas at the end of differentiation on day 30. There was considerable variability observed in cardiac differentiation efficiency among the different hPSC lines. The highest number of beating areas was found in the H7 and UTA.00106.hFF cell lines while UTA.04602.WT, UTA.00525.LQT2 and UTA.00112.hFF produced fewer beating areas (Figure 2B).

Two hPSC lines (H7 and UTA.00525.LQT2) produced the highest number of beating areas when they were cultured on a MEF feeder cell layer prior to differentiation and one hPSC line (UTA.00106.hFF) when cultured on an SNL feeder cell layer (Figure 2B). In the H7 cell line, the number of beating areas was significantly lower on Matrigel when compared to SNL (p = 0.002) or MEF (p<0.001) feeder cell layers. UTA.00525.LQT2 produced the highest number of beating areas when cultured on a MEF feeder cell layer, and the number was significantly higher that on Matrigel (p = 0.017) (Figure 2B). In the UTA.00106.hFF cell line, the number of beating areas was highest in cells cultured on an SNL feeder cell layer prior to differentiation (p<0.001). Taken together, three cell lines produced the highest number of beating areas when cultured on mouse feeder cell layers prior the differentiation. The UTA.04602.WT cell line was an exception, and the number of beating areas was significantly higher in cells taken from Matrigel cultures than from MEF feeder cell cultures (p = 0.015). The UTA.00112.hFF cell line had a very poor differentiation capacity and produced only a few beating areas overall, and there were no significant differences among the different culture conditions.

The highest number of Troponin T positive cells was found in cells originating on MEF feeder cell layers, while the number of Troponin T positive cells was lowest in cells originating on Matrigel (p = 0.012) (Figure 2C). The amount of Troponin T positive cells for each hPSC line is presented in Figure 2D. Although the total number of cytospin experiments was quite low, the results were consistent with the number of beating areas counted.

### The expression of developmental markers varies in cells cultured in different conditions

RNA samples were collected from undifferentiated cells at the beginning of cardiac differentiation (day 0) and from END-2 co-cultures on days 3, 6, 12 and 30 during two individual differentiation experiments of the H7, UTA.00112.hFF and UTA.04602.WT cell lines. The expression of the marker for pluripotent stem cells (*OCT-3/4*), the mesodermal markers (*T, brachyury homolog* (*Brachyury T*) and *NK2 homeobox 5* (*NKX2.5*)), and the endodermal marker (*Sex determining region Y-box 17* (*SOX-17*)) were analyzed in the samples.

**Figure 3:**
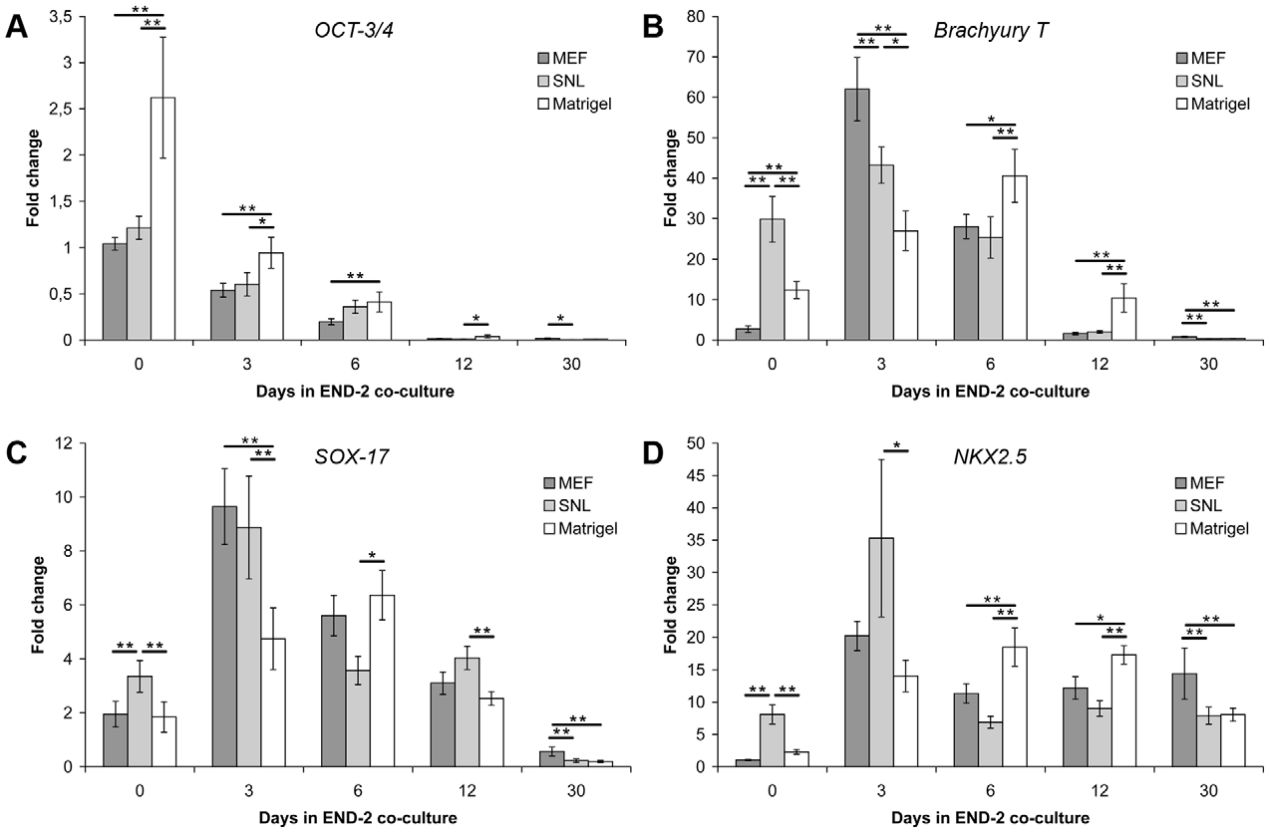
The expression profiles of *OCT-3/4*, *Brachyury T*, *SOX-17* and *NKX2.5* in END-2 co-cultures. A) The expression of *OCT-3/4* in END-2 co-cultures originating from Matrigel decreased slower than in MEF and SNL feeder cell layer. B) The expression of *Brachyury T* peaked on day 3 in END-2 co-cultures originating from MEF and SNL feeder cell layers, while from Matrigel the peak was delayed to day 6. C) The expression of *SOX-17* behaved in the same way than *Brachyury T* expression. *SOX-17* peaked on day 3 in END-2 co-cultures originating from MEF and SNL feeders, while in co-cultures originating from Matrigel, the *SOX-17* peak was delayed to day 6. D) The expression of *NKX2.5* was highest on MEF feeder cell layers in the end of END-2 co-culture. The data is collected from two individual differentiation experiments of H7, UTA.00112.hFF and UTA.04602.WT hPSC lines (n = 6 in all three conditions). Error bars show the standard error of the mean (SEM). ** p<0.01, * p<0.05.

*OCT-3/4* expression progressively declined during END-2 co-culture in all culture conditions (Figure 3A). On Matrigel, the *OCT-3/4* expression was significantly higher than on MEF and SNL feeder cell layers on day 0 (MEF vs. Matrigel, p = 0.003; and SNL vs. Matrigel, p = 0.009) and on day 3 (MEF vs. Matrigel, p = 0.004; SNL vs. Matrigel, p = 0.018). On day 6, the expression of *OCT-3/4* was significantly higher in cells taken from Matrigel than those from MEF feeder cell layers (p = 0.007). Thus, the expression of *OCT-3/4* persisted for a longer time in hPSCs cultured on Matrigel as opposed to MEF or SNL feeder cell layers (Figure 3A).

In cells originating on MEF or SNL feeder cell layers, the peak level of *Brachyury T* expression was observed on day 3, while cells originating on Matrigel showed peak *Brachyury T* expression later on day 6 (Figure 3B). The highest expression level of *Brachyury T* was observed on day 3 in cells cultured on a MEF feeder cell layer prior to differentiation (MEF vs. SNL, p = 0.004; MEF vs. Matrigel, p<0.001). On day 3, the expression of *Brachyury T* in cells originating on Matrigel was significantly lower than the expression level in cells cultured on MEF (p<0.001) or SNL (p = 0.016) feeder cell layers. On day 6 and 12, the expression of *Brachyury T* was significantly higher in cells cultured on Matrigel than those cultured on MEF (day 6, p  = 0.040; day 12, p = 0.001) or SNL (day 6, p = 0.009; day 12, p = 0.001) feeder cell layers prior to differentiation.

The expression of endodermal *SOX-17* was similar when compared to mesodermal *Brachyury T* expression (Figure 3C). The highest level of *SOX-17* expression was found in cells originating on MEF feeder cell layers on day 3. *SOX-17* peaked on day 3 in cells cultured on MEF and SNL feeder cell layers, whereas the peak level of *SOX-17* expression in cells cultured on Matrigel was observed on day 6. On day 3, *SOX-17* expression in cells originating on Matrigel was significantly lower than in cells cultured on MEF (p<0.001) or SNL (p = 0.001) feeder cell layers, while on day 6, *SOX-17* expression was significantly higher in cells originating on Matrigel than on an SNL feeder cell layer (p = 0.016).

The highest expression of *NKX2.5* was observed on day 3 in cells from SNL feeder cells, and it was significantly higher (p = 0.047) than expression levels in cells cultured on Matrigel. On day 12, the situation was just the opposite, in that the expression of *NKX2.5* was higher in cells originating on Matrigel than on MEF (p = 0.018) or SNL (p<0.001) feeder cell layers. On day 30, *NKX2.5* expression was significantly higher in cells originating on MEF than on SNL feeder cell layers or Matrigel (p<0.001 for both conditions).

### PSA-NCAM positive cells can be detected in all culture conditions

**Figure 4:**
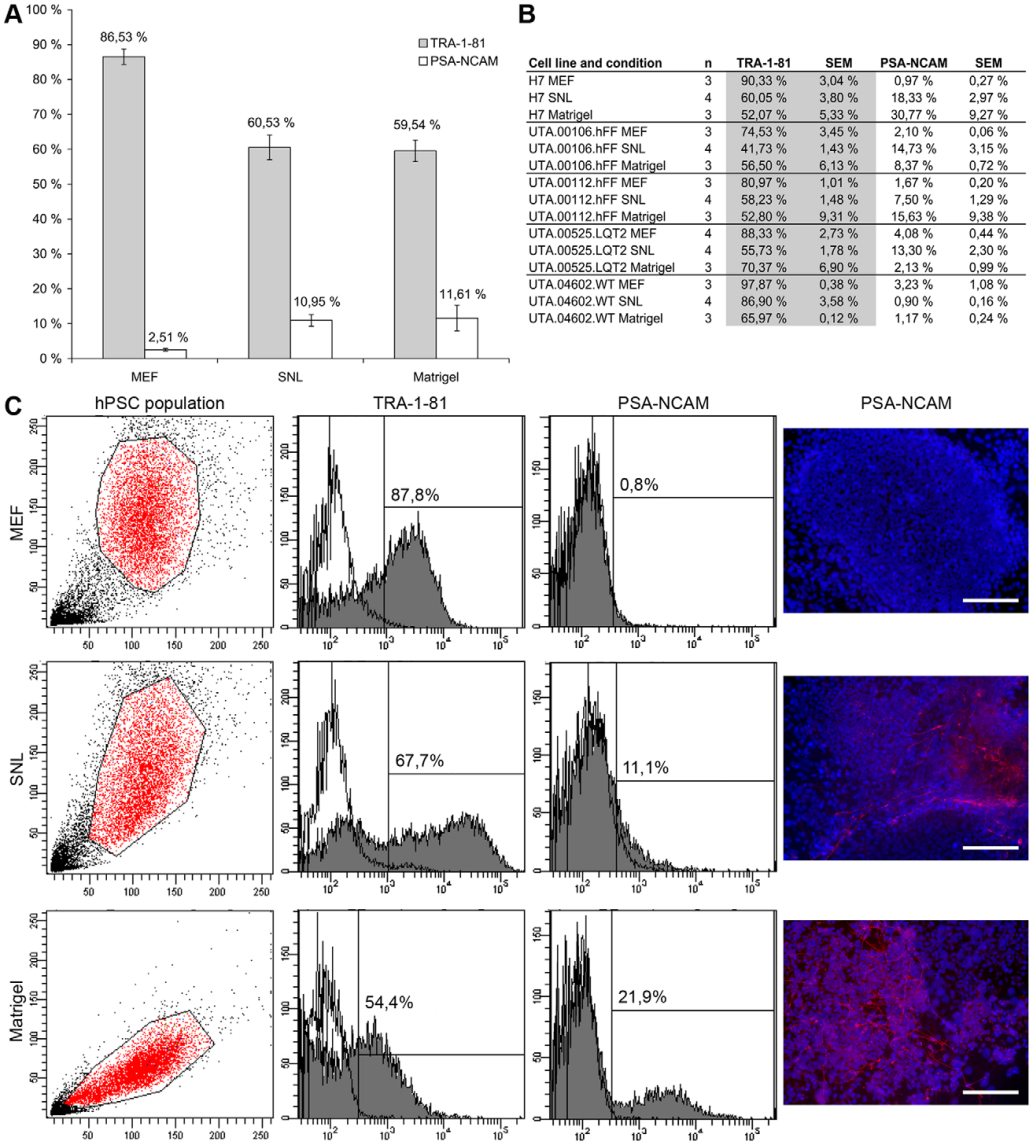
The amount of TRA-1-81 and PSA-NCAM positive cells in hPSC cultures. A) The amount of TRA-1-81 positive cells was higher and the expression of PSA-NCAM positive cells was lower in MEF feeder cultures than in SNL or Matrigel cultures. Columns show the average of TRA-1-81 and PSA-NCAM positive cells of all hPSC lines cultured in three different conditions (MEF n = 16, SNL n = 20 and Matrigel n = 15). Error bars show the standard error of the mean (SEM). B) The amount of TRA-1-81 and PSA-NCAM cells for each hPSC lines cultured in all three conditions. C) Examples of TRA-1-81 and PSA-NCAM expressions in H7 cell line in all three conditions. Dot plots show the determination of hPSC population and histograms show the percentage of TRA-1-81 and PSA-NCAM positive cells. Unstained cells were used for background determination (white). The highest amount of PSA-NCAM positive cells in immunocytochemical stainings were found on Matrigel. PSA-NCAM positive cells were not detected on MEF feeder cell cultures. Scale bars, 200 μm.

All five hPSC lines cultured in all three conditions were analyzed with cytometric analysis of pluripotency marker tumor-related antigen (TRA)-1-81 and polysialylated-neural cell adhesion molecule (PSA-NCAM), which is mainly expressed in embryonic and neonatal neural tissue. PSA-NCAM positive cells could be detected from all hPSC culture conditions (Figure 4A–B). The lowest amount of PSA-NCAM positive cells about 2.5% was found in MEF cultures, while in SNL and Matrigel cultures the amount of PSA-NCAM positive cells was about 11%. H7 and UTA.00112.hFF cell lines had the highest amount of PSA-NCAM positive cells in Matrigel cultures, UTA.00106.hFF and UTA.00525.LQT2 in SNL cultures and UTA.04602.WT cell line in MEF cultures. In general, the amount of TRA-1-81 positive cells positive cells correlated with the amount of PSA-NCAM positive cells. It was highest in MEF cultures and lowest in SNL and Matrigel cultures (Figure 4A). In all hPSC lines, the amount of TRA-1-81 positive cells was highest in MEF cultures indicating the superiority of MEF feeder layer over SNL feeders and Matrigel in maintaining the pluripotency of hPSCs. PSA-NCAM positive cells were not detected in MEF cultures in immunocytochemical stainings, while on SNL feeder cell layers and on Matrigel cultures PSA-NCAM positive cells were detected. Example of TRA-1-81 and PSA-NCAM positive cells and immunocytochemical staining with PSA-NCAM antibody are presented in Figure 4C for H7 cell line. H7 cell line is widely used in cardiac differentiation experiments and it had the best cardiac differentiation efficiency of all hPSC lines used in this experiment.

### Neural cells are more abundant in Matrigel cultures than in feeder cultures

**Figure 5:**
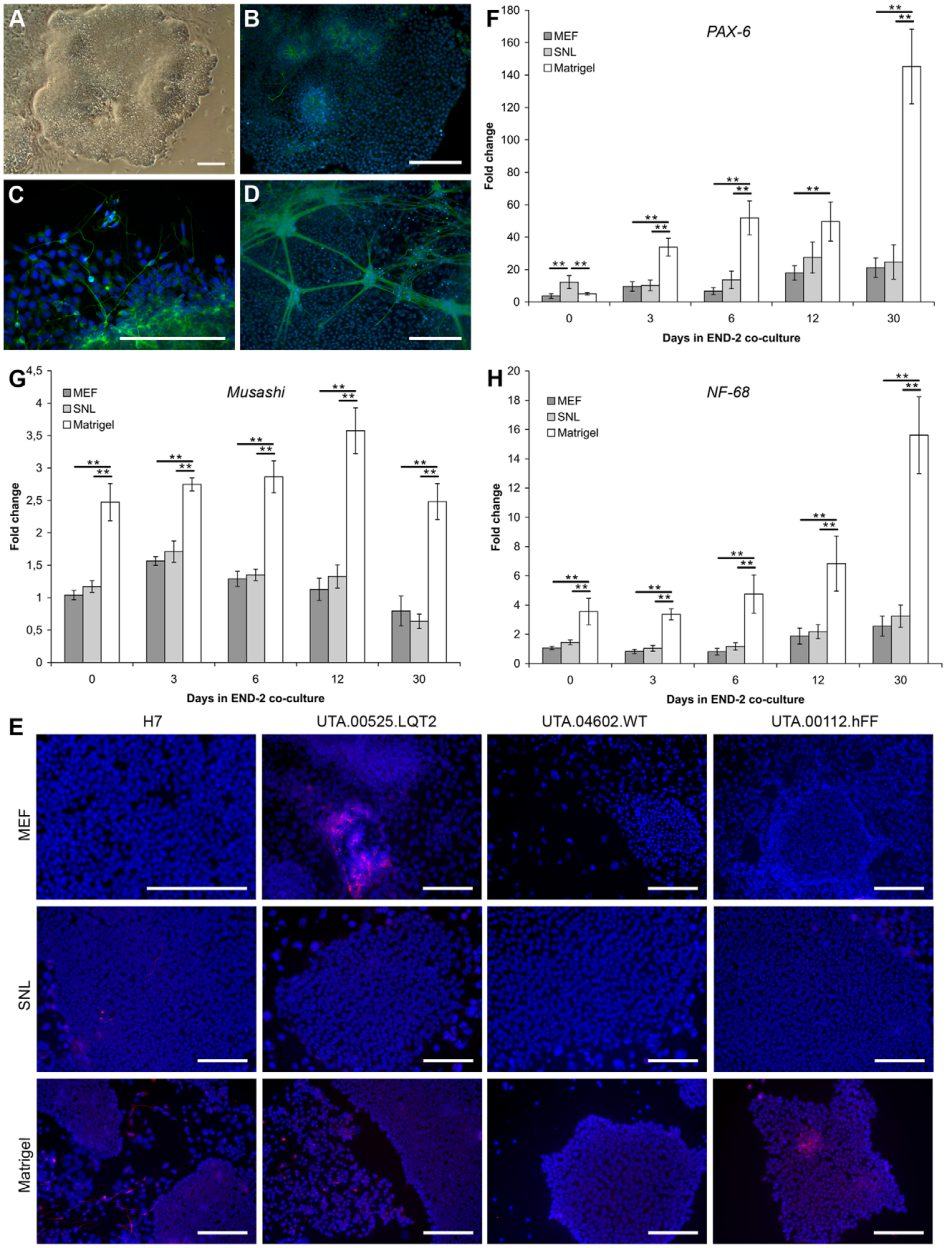
The expression of neural markers was highest in hPSCs originating from Matrigel. A) H7 cell line was hard to adapt on Matrigel combined with mTeSR1 medium. At first, the neural differentiation was observed primarily along the edges of the colonies on Matrigel in mTeSR1 medium. Finally, uneven neural rosette-like structures were formed in the colonies and the cell line was lost. B) Uneven neural rosette-like structures found in colonies of H7 cell line cultured on Matrigel in mTeSR1 medium stained with MAP-2. C) MAP-2 expressing neural-like cells and structures were found on Matrigel in mTeSR1 medium in all hPSC lines. Representative image of UTA.04602.WT cell line. D) MAP-2 expressing neural structures appeared also in END-2 co-cultures when hPSCs originated from Matrigel and mTeSR1 cultures with all hPSC lines. Representative image of H7 cell line. Scale bars, 200 μm. E) The highest amount of MAP-2 positive cells were found in Matrigel cultures. Scale bars, 200 μm. The expression of *PAX-6 (F), Musashi (G)* and *Neurofilament (NF-68)* (H) in END-2 co-cultures was significantly higher in cells originating from Matrigel and mTeSR1 medium than from MEF or SNL feeder cell layers almost in all time points. The data is collected from two individual differentiation experiments of H7, UTA.00112.hFF and UTA.04602.WT hPSC lines (n = 6 in all three conditions). Error bars show the standard error of the mean (SEM). ** p<0.01, * p<0.05.

On Matrigel matrix combined with mTeSR1 medium, hPSCs tended to differentiate into neural-like cells, which was observed primarily along the edges of the undifferentiated hPSC colonies (Figure 5A). Neural-like cells were observed occasionally in all cell lines cultured on Matrigel in mTeSR1 medium and two hPSC lines H7 and UTA.00112.hFF were hard to adapt on Matrigel because of the neural differentiation. The H7 cell line was hard to maintain on Matrigel in mTeSR1 medium, and as a result of differentiation into neural-like cells, the H7 cell line was lost after 7 passages in culture. At first, there were only a few neural-like cells observed in the culture, but the number of neural cells expanded during the culture period, even though the differentiated areas were carefully removed before passaging the cells. Finally, only colonies with MAP-2 expressing, rosette-like structures (Figure 5A–B) and neural-like cells (Figure 5C) were observed in the cultures. H7, UTA.00112.hFF, UTA00525.LQT2 and UTA.04602.WT cell lines cultured in all three culture conditions were characterized by immunocytochemical staining with MAP-2. Only a few MAP-2 positive cells were found in SNL and MEF cultures, but none were found to the same extent as in cells cultured on Matrigel in mTeSR1 medium (Figure 5E). On SNL feeders, the only MAP-2 positive cells were found from H7 cell line and on MEF feeders from UTA.00525.LQT2 cell line (Figure 5E).

The expression of ectodermal *PAX-6, Musashi* and *Neurofilament 68* (*NF-68*) was measured by q-RT-PCR in cells cultured for 0, 3, 6, 12 and 30 days in END-2 co-cultures (Figure 5F–H). Samples were collected during two individual differentiation experiments of the H7, UTA.00112.hFF and UTA.04602.WT cell lines. The expression of *PAX-6* increased in all conditions from day 0 to day 30 (Figure 5F). However, the expression of *PAX-6* in cells originating from Matrigel was significantly higher than on MEF of SNL feeder layers on day 3 (Matrigel vs. MEF p<0.001, Matrigel vs. SNL p<0.001), 6 (Matrigel vs. MEF p<0.001, Matrigel vs. SNL p<0.001) and 30 (Matrigel vs. MEF p<0.001, Matrigel vs. SNL p<0.001). On day 30 the expression of *PAX-6* was about 7 times higher in cells originating from Matrigel than from feeder cell layers. The expression of *Musashi* (Figure 5G) and *NF-68* (Figure 5H) remained stable in cells originating on MEF and SNL feeder cell layers. The expression of *Musashi* was significantly higher in all time points in cells originating from Matrigel than from MEF or SNL feeder cell layers (p<0.001) (Figure 5G). There were no significant differences between MEF and SNL feeder cell layers in the expression of *Musashi*. The expression of *NF-68* increased steadily from day 0 to day 30 (Figure 5H): day 0 (Matrigel vs. MEF, p<0.001; Matrigel vs. SNL, p = 0.003); day 3 and day 6 (p<0.001 for both conditions); day 12 (Matrigel vs. MEF, p = 0.002; Matrigel vs. SNL, p = 0.005) and day 30 (p<0.001 for both conditions). Interestingly, MAP-2 expressing neural-like cells and nerve bundles could also be detected in END-2 co-cultures when hPSCs were cultured on Matrigel prior to differentiation (Figure 5D). These structures were not found in END-2 co-cultures originating from mouse feeder cell layers.

## Discussion

Several studies have evaluated the pluripotent and undifferentiated growth of hPSCs in serum-, xeno- and feeder cell-free conditions [26]–[29]. Previously we have shown that MEF feeder cells support cardiac differentiation better than human foreskin fibroblast (hFF) feeder cells [30]. In this study, we cultured hPSCs with three different culture methods: on MEF [1] and on SNL [31] feeder cell layers combined with conventional ko-SR and bFGF containing stem cell culture medium and on Matrigel with mTeSR1 medium [9], and evaluated the influence of the culture method on the cardiac differentiation potential of hPSCs. The cardiac differentiation efficiency varied between hPSC lines and separate differentiation experiments. In general our results suggest that hPSCs cultured on MEF and SNL feeder cell layers together with conventional stem cell culture medium are more prone to cardiac differentiation than hPSCs cultured on Matrigel combined with mTeSR1 medium, with one cell line UTA.04602.WT as an exception.

In this study, all hPSC lines differentiated into cardiomyocytes, but the differentiation efficiency varied considerably depending on the individual cell line, separate differentiation experiments and on the conditions under which the cell lines had been cultured prior to differentiation. Each hPSC line has a unique gene expression profile and thus vary in their cardiac differentiation potential [32]. In addition, it has been shown that hPSC lines change over time in culture due to genomic alterations [20]. For example enzymes used in hPSC passaging might have an effect to the pluripotent growth of hPSCs [22]. In our study the same enzymes which were used as in the original publications of the culture methods. The highest cardiac differentiation efficiency was observed in cells cultured on MEF or SNL feeder cell layers together with stem cell culture medium and the lowest in cells cultured on Matrigel in mTeSR1 medium. However, one cell line UTA.04602.WT was an exception and produced the highest amount of beating areas when cultured previously on Matrigel in mTeSR1. We characterized the starting population of hPSCs in all three conditions with flow cytometric analysis of TRA-1-81 and PSA-NCAM to determine the amount of pluripotent cells and if more cells were already committed to ectodermal lineages in some culture condition. The amount of PSA-NCAM positive cells was lower and the amount of TRA-1-81 positive cells was higher on MEF feeder cell cultures than in SNL feeder cell cultures and Matrigel cultures, indicating the superiority of MEF feeders in maintaining the pluripotency of hPSCs.

Mouse feeder cells express high levels of Activin A and low levels of bone morphogenetic protein 4 (BMP-4), which together have been reported to induce cardiac differentiation [33]–[35]. Normally, the peak *Brachyury T* expression is observed on day 3 in END-2 co-cultures [36]. Previously, we showed that peak *Brachyury T* expression was delayed to day 6 in cells cultured on hFF feeder cell layers [30]. The delayed peak of *Brachyury T* expression has been shown to lead to poor cardiac differentiation efficiency [37]. In this study, the expression peak of *Brachyury T* in cells cultured on Activin A expressing MEF and SNL feeder cells prior the differentiation was observed on day 3 and the peak *Brachyury T* expression was delayed to day 6 in cells cultured on Matrigel in mTeSR1 medium. Furthermore, endodermal *SOX-17* expression has been shown to enhance the cardiac differentiation and should peak on the same day on day 3 as *Brachyury T* [36], [37]. The peak *SOX-17* expression was observed on day 3 in co-cultures originating from MEF and SNL feeder cell layers while the expression peak of *SOX-17* in END-2 co-cultures originating on Matrigel was delayed to day 6 in the same way than the expression peak of *Brachyury T*. The delayed expression of *Brachyury T* and *SOX-17* might indicate that hPSCs grown on Matrigel in mTeSR1 medium need more time to the initiation of the differentiation to mesodermal lineages. However, we counted the Troponin T positive cells 20 days and the beating areas 30 days after the initiation of END-2 co-culture and all our data suggest that hPSCs cultured on MEF and SNL feeder cell layers were more prone to cardiac differentiation than hPSCs cultured on Matrigel. The delayed expression of *Brachyury T* and *SOX-17* may be one reason for differences in cardiac differentiation efficiencies. However, to explain this phenomenon additional experiments are required.

Matrigel matrix together with mTeSR1 medium is widely used in different laboratories, and several research groups have reported this culture condition to maintain the undifferentiated growth of hPSCs [8], [9], [26], [27], [38]. In these studies, the pluripotency of hPSCs was confirmed by teratoma and EB formation and analysis of the expression of markers specific to all three germ layers present in these structures. Recently, Hudson and co-workers adapted hESCs to Matrigel in mTeSR1 medium and demonstrated that passaging the cells as single-cells prior to their cardiac differentiation reduced the heterogeneity of the cell population and enhanced cardiac differentiation [39]. However, they cultured hESCs on Matrigel in mTeSR1 medium for only one passage. To our knowledge, this is the first study reporting the long-term effects of this culture method on cardiac differentiation.

In this study, more MAP-2 positive cells could be detected in long-term culture of hPSCs on Matrigel matrix together with mTeSR1 medium than in MEF and SNL feeder cell cultures. The expression of *PAX-6, Musashi* and *NF-68* increased during END-2 co-culture in cells cultured on Matrigel in mTeSR1 medium prior to differentiation, and the emergence of neuronal cells in END-2 co-cultures was confirmed by MAP-2 staining. Beqqali and co-workers have reported that the expression of ectodermal genes in the END-2 co-culture method is low when compared to endodermal and mesodermal genes [36]. Erceg and co-workers have described the efficient differentiation of hESCs into neural lineages, using a mixed ECM protein (collagen IV, vitronectin and fibronectin) coating together with TeSR1 [8] medium, in which human serum albumin was replaced by Voluven [40]. In addition, laminin is one of the major constituents of Matrigel [41], [42], and both Matrigel [43] and laminin are widely used as a matrix when generating neuronal cells and their derivatives from hPSCs [44]–[46]. In fact, Ma and co-workers tested five substrates, including poly-D-lysine, fibronectin, laminin, collagen and Matrigel; and they observed that laminin and laminin-rich Matrigel significantly enhanced directed differentiation into neural progenitors and neurons [44]. Axell and co-workers reported that hESCs cultured on Matrigel were more efficient source of neural progenitors than hESCs cultured on MEF feeder cell layers [45]. They assumed that this was due to the fact that cells cultured on Matrigel were already adapted to feeder cell-free cultures for 6–12 passages before their transfer to neural differentiation conditions. In addition to the neural differentiation inducing properties of Matrigel, mTeSR1 medium contains high concentrations of bFGF, transforming growth factor beta (TGF-β), gamma aminobutyric acid (GABA), pipecolinic acid and lithium chloride [9]. Further, mTeSR1 medium contains a higher concentration of insulin (0.023 g/l) than conventional stem cell culture medium (0.01 g/l) and insulin has been suggested to inhibit cardiac differentiation in END-2 co-cultures and actually redirecting the differentiation from cardiac mesoderm and endoderm into neuroectoderm [47]. The concentration of bFGF in mTeSR1 medium (100 ng/ml) is extremely high when compared to basic stem cell culture medium (4 ng/ml), and mouse feeder cells do not express bFGF [33]. In neural precursor media, the concentration of bFGF is normally 10–20 ng/ml [43], [48]–[51] and bFGF has been found to play a role in the derivation, proliferation and maintenance of the neural progenitor state [43], [45], [52]. Thus, it is possible that, together with the laminin found in Matrigel, some of these factors: bFGF, TGF-β, GABA, pipecolinic acid, lithium chloride and insulin somehow induce more neural differentiation of hPSCs than differentiation to mesoendodermal lineages.

In our study, the expression of *OCT-3/4* was significantly higher in hPSCs cultured on Matrigel in mTeSR1 medium than on MEF or SNL feeder cell layers. Retention of *OCT-3/4* expression has been observed in neural progenitor populations, and the rapid loss of *OCT-3/4* expression during neural progenitor differentiation has been reported to induce hPSCs to develop into flattened extraembryonic cells rather than neural cells [45], [53], [54]. In fact, prolonged expression of *OCT-3/4* could be required for the neural differentiation while the rapid downregulation of *OCT-3/4* may be required to promote the formation of primitive endoderm that is essential for mesodermal differentiation [53]. Our results suggest that hPSCs cultured on Matrigel in mTeSR1 medium are more prone to neural lineages as to mesoendodermal lineages after long-term culture, and thus, the cardiac differentiation efficiency remains low.

Here, we have studied the effects of hPSC culture methods on the cardiac differentiation efficiency of hPSCs. Five hPSC lines cultured in three different culture conditions differentiated into beating cardiomyocytes, but the differentiation efficiency varied depending on the cell line, the specific differentiation experiment conducted and most importantly on the different culture conditions used. Mouse feeder cell layers (MEF and SNL) were found to be superior to the Matrigel matrix used together with mTeSR1 medium in inducing cardiac differentiation with one cell line as an exception out of all five hPSC lines. In fact, more MAP-2 expressing cells could be found in Matrigel and mTeSR1 cultures than from MEF and SNL feeder cell cultures. Our suggestion is that, in addition to the specific differentiation method, the hPSC culture method should also be optimized when differentiating hPSCs into specific lineages. In addition, the combination of culture conditions and differentiation conditions might be important for cardiac differentiation. At least our results show that mouse feeder cells should be used instead of Matrigel and mTeSR1 medium in combination of END-2 differentiation method.

## Materials and Methods

### Ethical issues

The study was conducted in accordance with the Ethics Committee of Pirkanmaa Hospital District to establish, culture and differentiate hESC and hiPSC lines (R08070, R05116). Skin biopsies for hiPSC establishment were received from the Heart Center, Tampere University Hospital. Patients donating skin biopsies signed an informed consent form after receiving both an oral and written description of the study.

### Cell lines and cell culture

The hESC line H7 (46, XX) (WiCell Research Institute, Madison, WI, USA) [1] and four hiPSC lines including UTA.00112.hFF (46, XY) and UTA.00106.hFF from hFFs, and UTA.04602.WT (46, XX) and UTA.00525.LQT2 (46, XY) from adult human dermal fibroblasts were used in this study. hiPSC lines were reprogrammed with four retroviral vectors (*SOX-2, OCT-3/4, KLF4* and *C-MYC*) as described previously [55], [56]. The UTA.00106.hFF cell line was found to be karyotypically abnormal with inversion in chromosome 12 (46, XY inv(12)). All five cell lines were cultured for at least for 14 passages at +37°C and 5% CO_2_ in three different culture conditions, as described below.

All hPSC lines used in this study were normally cultured on mitomycin C treated MEF feeder cell layers (26000 cells/cm^2^) (Millipore Corporate, Billerica, MA, USA) in stem cell culture medium consisting of ko-DMEM (Invitrogen, Carlsbad, CA, USA) supplemented with 20% ko-SR (Invitrogen), 1% non-essential amino acids (NEAA, Cambrex Bio Science Inc., Walkersville, MD, USA), 2 mM GlutaMax (Invitrogen), 50 U/ml penicillin/streptomycin (Lonza Group Ltd, Basel, Switzerland), 0.1 mM 2-mercaptoethanol (Invitrogen) and 4 ng/ml bFGF (R&D Systems Inc., Minneapolis, MN, USA). The medium was changed three times per week, and the cells were passaged enzymatically onto a new MEF feeder cell layer once per week. The MEF feeder cell layer was removed manually with a pipette tip before detaching the hPSC colonies with 1 mg/ml Collagenase IV (Invitrogen).

All hPSC lines were cultured on irradiated (40 Gy) SNL 76/7 (HPA Culture Collections, Salisbury, UK) feeder cell layers (29000 cells/cm^2^) in stem cell culture medium. The medium was changed 6 times per week and hPSCs were passaged mostly in every five days (range 4–7 days) onto new SNL feeder cell layers. Before passaging, SNL feeder cells were removed with the previously described CTK solution [56] with a minor modification: ko-SR was replaced by stem cell culture medium without bFGF. CTK solution consisted of 10% Trypsin (10x, Lonza), 0.1 mg/ml Collagenase IV (Invitrogen), 0.001 M CaCl_2_ and 20% stem cell culture medium in H_2_O. After CTK treatment, the remaining SNL feeder cells were carefully rinsed with ko-DMEM (Invitrogen), and colonies were scraped into stem cell culture medium with a pipette tip and plated onto new SNL feeder cell layers.

All hPSC lines were cultured on hESC-qualified Matrigel (BD Biosciences, Franklin Lakes, NJ, USA) in mTeSR1 medium (StemCell Technologies Inc., Vancouver, Canada) supplemented with 50 U/ml penicillin/streptomycin (Lonza). MEF feeder cell layer was manually removed as described above; colonies were scraped into mTeSR1 medium and plated onto Matrigel-coated 6-well culture plates. Plates were coated with Matrigel for at least 1 hour at room temperature (RT) following manufacturer's instructions. mTeSR1 medium was changed 6 times per week, and the cells were passaged using 1 mg/ml dispase (Invitrogen) mostly in every 5 days (range 4–6 days). Differentiated areas were carefully removed before passaging.

The growth of hPSC lines in different conditions was monitored daily under Nikon Eclipse TS100 phase contrast microscope (Nikon Instruments Europe B.V. Amstelveen, The Netherlands) and pictured with Altra-Cell-D-Bundle camera (Olympus Corporation, Tokyo, Japan).

### Cardiac differentiation

Differentiation experiments were performed 2 to 6 times in hPSC lines cultured in all three conditions. hPSC lines and their passages in 6 separate differentiation experiments are presented in Table 1 and the cardiac differentiation experiments are outlined in Figure 1A. Altogether differentiation experiments were performed 18 times from MEF feeder cell layers and 15 times from both SNL feeder cell layers and Matrigel. All differentiation experiments with the UTA.00106.hFF cell line were performed with karyotypically abnormal (46, XY inv(12)) cells. To initiate cardiac differentiation, hPSCs were co-cultured in 12-well culture plates with Mitomycin C (Sigma-Aldrich, St. Louis, MO, USA) treated END-2 cells (50000 cells/cm^2^), which were a kind gift from Professor Mummery (Humbrecht Institute, Utrecht, The Netherlands) [57]. MEF feeder cell layers were removed manually, and SNL feeder cells enzymatically with CTK solution before differentiation. Cell colonies cultured in all three conditions were detached with a cell scraper or pipette tip. Approximately 30 colony pieces per well were transferred onto END-2 cells in stem cell culture medium without ko-SR or bFGF and supplemented with 3 mg/ml ascorbic acid (Sigma-Aldrich). Medium was changed after 5, 8 and 12 days of culturing. After 15 days of culturing, 10% ko-SR was included and ascorbic acid was excluded from the culture medium; subsequently, the medium was changed three times per week.

### Cardiac differentiation efficiency

Cardiac differentiation efficiency was determined by cytospin analysis on day 16–21 (herein on day 20) and by counting the number of beating areas on day 28–35 (herein day 30). Cytospin analysis was performed and beating areas were counted from all five hPSC lines cultured in all three conditions. The total number of wells from which the beating areas were counted is presented in Table 1. For cytospin analysis, the whole differentiating pool of cells from three wells (A1, B1 and C1), and a replicate sample of all cells from another three wells (A2, B2 and C2) were treated with trypsin (Lonza) at 37°C for 45 minutes. After the incubation, the aggregates were pipetted into a single-cell suspension and resuspended in EB medium, consisting of ko-DMEM supplemented with 20% fetal bovine serum (FBS, PAA Laboratories GmbH, Pasching, Austria), 1% NEAA (Cambrex Bio Science), 2 mM GlutaMax (Invitrogen) and 50 U/ml penicillin/streptomycin (Lonza). Approximately 1×10^6^ cells were centrifuged at 800–1000 rpm for 5 minutes onto polysine slides (Thermo Scientific, Rochester, NY) using the cytospin system (Sakura Finetek, Alphen aan den Rijn, The Netherlands). Adherent cells on slides were fixed with 4% paraformaldehyde (Sigma-Aldrich) at RT for 20 minutes, permeabilized and blocked with 0.1% Triton X-100 (Sigma-Aldrich), 1% bovine serum albumin (BSA, Sigma-Aldrich) and 10% normal donkey serum (Sigma-Aldrich) in phosphate-buffered saline (PBS, Lonza) for 45 min at RT, and stained with mouse or goat anti-cardiac Troponin T primary antibodies (Table S1) diluted in 1% normal donkey serum, 0.1% TritonX-100, and 1% BSA in PBS (Lonza) over night at +4°C. The next day, the cells were probed with Alexa Fluor 568 secondary antibody (Invitrogen) diluted in 1% BSA (Sigma-Aldrich) in PBS (Lonza) for 1 h at RT in the dark. Finally, cells were mounted with Vectashield (Vector Laboratories Inc., Burlingame, CA, USA) containing 40,6-diamidino-2-phenylindole (DAPI) for nuclear staining, and the percentage of Troponin T positive cells versus the total cell number was determined. Counted areas were randomly selected in the DAPI channel using 20 x magnification, and a total of 1500 cells was counted. Cells were pictured with Olympus IX51 phase contrast microscope with fluorescence optics and Olympus DP30BW camera (Olympus Corporation).

### Dissociation of beating areas and immunocytochemistry

Beating areas were cut out manually and dissociated into a single-cell suspension using Collagenase A (Roche Diagnostics GmbH, Mannheim, Germany) treatment, as previously described [58]. Dissociated cells were plated onto 0.1% gelatin coated 24-well plates in EB medium.

The undifferentiated growth and neural differentiation of hPSCs under different culture conditions was judged by the morphology of the cells. The morphologic characterization was confirmed by immunocytochemical stainings for hPSCs cultured at least for 14 passages in three different culture conditions. hPSC colonies were stained with primary antibodies specific for undifferentiated hPSCs including Nanog, OCT-3/4 and SSEA-4. Neural progenitor cells and neuronal cells were detected from undifferentiated cultures by staining cells for PSA-NCAM and MAP-2. Dissociated cardiomyocytes were stained for connexin-43, α-actinin, Troponin T and MHC. The primary antibodies are summarized in Table S1 and staining was performed as described above. All Alexa Fluor 568 or 488-conjugated secondary antibodies were from Invitrogen.

### In vitro analysis of pluripotency

The pluripotency of the H7, UTA.00106.hFF and UTA.00525.LQT2 cell lines cultured in all three culture conditions was verified by the formation of EBs. To form EBs, feeder cells were removed mechanically (MEF) or enzymatically (SNL), and hPSCs were scraped with a cell scraper and placed into suspension culture in EB medium. Media was changed every 2 to 3 days, and EBs were cultured for 5 weeks. Total RNA was extracted from EBs and 200 ng of cDNA was transcripted. The expression of the three germ layers, ectoderm (*Paired box 6* (*PAX-6*) and *SRY-box 1* (*SOX-1*)), endoderm (*Alpha-fetoprotein* (*AFP*) and *SOX-17*) and mesoderm (*α-cardiac actin* and *Kinase insert domain receptor* (*KDR*)) was studied in the EBs using RT-PCR primers. *β-actin* was used as housekeeping control. Primer sequences are presented in Table S2.

### Quantitative-RT-PCR

Quantitative RT-PCR was performed on H7, UTA.00112.hFF and UTA.04602.WT cell samples collected from the fourth and sixth differentiation experiments (Table 1). Each of two replicate samples were collected from two wells of co-cultures and lysed into RA1 buffer supplemented with β-mercaptoethanol at time points of 3, 5–6, 12–13 and 28–35 days, herein reported as 3, 6, 12 and 30 days. Undifferentiated cells were used as day 0 samples. Samples were stored at −70°C until the total-RNA was extracted with the NucleoSpin® RNA II kit, which included DNAase treatment (Macherey-Nagel, Duren, Germany) as described in the manufacturer's instructions. The concentration and quality of RNA was measured using a NanoDrop 1000 spectrophotometer (NanoDrop Technologies, Wilmington, DE). Biological replicates were pooled into one sample during cDNA transcription, and 250 ng of RNA from both biological replicates (totaling 500 ng) were transcribed into cDNA in a total volume of 20 μl with a High-Capacity cDNA Reverse-Transcription kit (Applied Biosystems, Foster City, CA, USA) in the presence of RiboLock RNase inhibitor (Thermo Scientific). The expression of *Brachyury T, NKX2.5* and *SOX-17* were studied with SYBR chemistry and *Ribosomal protein large p0* (*RPLP0*), *OCT-3/4, PAX-6, Musashi* and *NF-68* were studied with Taqman chemistry. The PCR reaction for SYBR primers consisted of 1 μl of cDNA at a 1∶3 dilution, 14 μl of 2× SYBR green PCR mastermix (Applied Biosystems) and 300 nM of each primer. The following Taqman assays were used: NM_053275.3 for *RPLP0*, Hs00999632_g1 for *OCT-3/4* (*POU5F1*), Hs00240871_m1 for *PAX-6*, Hs01045894_m1 for *Musashi* and Hs00196245_m1 for *NF-68*. SYBR primer sequences are presented in Table S2. All samples were analyzed in triplicate, C_τ_ values were determined, and the fold changes were calculated by the 2^−ΔΔCτ^ method [59]. The data were normalized to the expression of the endogenous control *RPLP0*. The average of d0 samples from MEF feeder cell layers were used as a calibrator.

### Flow cytometric analysis

All five hPSC lines cultured in all three conditions were analyzed by flow cytometry using antibodies against TRA-1-81-FITC (BD Biosciences) and PSA-NCAM-APC (Miltenyi Biotec, Teterow, Germany). Samples were collected from one day before or same day as passaging was done. MEF and SNL feeder cells were removed prior the sample collections. FITC mouse IgM isotype control antibody (BD Biosciences) was used as isotype control. Labeled hPSCs were analyzed using BD FACSAria™ (BD Biosciences). The samples were analyzed as duplicates and the acquisition was set for 10000 events per sample. The data were analyzed using FACSDiva Software version 6.1.3 (BD Biosciences).

### Statistical analysis

The number of beating areas is presented as the mean value over all differentiation experiments; error bars represent the standard error of the mean (SEM). The number of the Troponin T positive cells in one well detected in cytospin analysis and are presented in scatter blots. Quantitative RT-PCR data are presented as the mean value ± SEM. For beating areas, cytospin and q-RT-PCR results statistical significance was determined using one-way ANOVA with Bonferroni's correction for multiple comparisons. Results were confirmed by Poisson regression analysis. However, for the sake of simplicity, only the ANOVA results are reported. A p-value <0.05 was considered statistically significant.

## Supporting Information

Table S1 **Primary antibodies used in this study.**
(DOC)

Table S2 **RT-PCR and quantitative RT-PCR primers used in this study.**
(DOC)
