# Supplementary material for: Culture Conditions Affect Cardiac Differentiation Potential of Human Pluripotent Stem Cells
Source: PLoS One. 2012 Oct 31;7(10):e48659. doi: 10.1371/journal.pone.0048659 (PMC3485380; doi:10.1371/journal.pone.0048659)
Supplement: Table S1 — Primary antibodies used in this study. (DOC) [file pone.0048659.s001.doc]

**Table S1.** Primary antibodies used in this study.

| **Primary antibody** | **Target** | **Origin** | **Dilution** | **Manufacturer** |
| --- | --- | --- | --- | --- |
| OCT-3/4 | Undifferentiated hPSCs | goat | 1:400 | R&D Systems |
| Nanog | Undifferentiated hPSCs | goat | 1:200 | R&D Systems |
| SSEA-4 | Undifferentiated hPSCs | mouse | 1:200 | Santa Cruz Biotechnology |
| MAP-2 | Neuronal cells | rabbit | 1:400 | Millipore |
| PSA-NCAM | Neuronal cells | mouse | 1:1000 | Millipore |
| α-actinin | Cardiomyocytes | mouse | 1:1500 | Sigma |
| Troponin T | Cardiomyocytes | goat | 1:2000 | Abcam |
| Troponin T | Cardiomyocytes | mouse | 1:500 | Abcam |
| Myosin ventricular heavy chain α/β | Cardiomyocytes | mouse | 1:100 | Millipore |
