# Supplementary material for: Culture Conditions Affect Cardiac Differentiation Potential of Human Pluripotent Stem Cells
Source: PLoS One. 2012 Oct 31;7(10):e48659. doi: 10.1371/journal.pone.0048659 (PMC3485380; doi:10.1371/journal.pone.0048659)
Supplement: Table S2 — RT-PCR and quantitative RT-PCR primers used in this study. (DOC) [file pone.0048659.s002.doc]

**Table S2.** Sequences for q-RT-PCR and RT-PCR primers used in this study.

| **Gene** | **GenBank ID** | **Target** | **Forward primer** | **Size (bp)** |
| --- | --- | --- | --- | --- |
| **q-RT-PCR** |  |  |  |  |
| *NKX2.5* (*NK2 homeobox 5*) | 1482 | Early cardiogenesis | **F:** CAAGGACCCTAGAGCCGAAA | 58 |
|  |  |  | **R:** CAGCTCCACCGCCTTCTG |  |
| *Brachyury T* (*T, brachyury homolog*) | 6862 | Mesoderm | **F:** TGCTTCCCTGAGACCCAGTT | 67 |
|  |  |  | **R:** TTTTAAGAGCTGTGATCTCCTCGTT | |
| *SOX-17* (*SRY (sex determining region Y)-box 17*) | 64321 | Definitive endoderm | **F:** GGCGCAGCAGAATCCAGA | 60 |
|  |  |  | **R:** CCACGACTTGCCCAGCAT |  |
| **RT-PCR** |  |  |  |  |
| *PAX-6* (*Paired box 6*) | 5080 | Ectoderm | **F:** AACAGACACAGCCCT | 274 |
|  |  |  | **R:** CGGGAACTTGAACTG |  |
| *SOX-1* (*SRY (sex determining region Y)-box 1*) | 6656 | Ectoderm | **F:** CACAACTCGGAGATC | 171 |
|  |  |  | **R:** GTCCTTCTTGAGCAG |  |
| *AFP* (*Alpha-fetoprotein*) | 174 | Endoderm | **F:** GCTGGATTGTCTGCA | 216 |
|  |  |  | **R:** TCCCCTGAAAAT |  |
| *SOX-17* (*SRY (sex determining region Y)-box 17*) | 64321 | Endoderm | **F:** CGCACGGAATTTGAA | 166 |
|  |  |  | **R:** CACACGTCAGGATAG |  |
| *α-cardiac actin* (*Actin, alpha, cardiac muscle 1*) | 70 | Mesoderm | **F:** GGAGTTATGGTGGGTATGGGTC | 486 |
|  |  |  | **R:** AGTGGTGACAAAGGAGTAGCCA |  |
| *KDR* (*Kinase insert domain reseptor*) | 3791 | Mesoderm | **F:** GTGACCAACATGGAGTCGTG | 218 |
|  |  |  | **R:** TGCTTCACAGAAGACCATGC |  |
| *β-actin* (*Actin, beta*) | 60 | Housekeeping control | **R:** GTCTTCCCCTCCATC | 302 |
|  |  |  | **R:** GGGGTGTTGAAGGTC |  |
